# Implementing TMLE in the presence of a continuous outcome

**DOI:** 10.1177/26320843231176662

**Published:** 2023-05-25

**Authors:** Hanna A Frank, Mohammad Ehsanul Karim

**Affiliations:** 1 School of Population and Public Health, 8166 University of British Columbia, Vancouver, BC, Canada; 2 Centre for Health Evaluation and Outcome Sciences, 8166 University of British Columbia, Vancouver, BC, Canada

**Keywords:** Causal inference, machine learning, targeted maximum likelihood estimation

## Abstract

In a real-world observational data analysis setting, guessing the true model specification can be difficult for an analyst. Unfortunately, correct model specification is a core assumption for treatment effect estimation methods such as propensity score methods, G-computation, and regression techniques. Targeted maximum likelihood estimation (TMLE) is an alternative method that allows the use of data-adaptive and machine learning algorithms for model fitting. TMLE therefore does not require strict assumptions about the model specification but preserves the validity of the inference. Multiple studies have shown that TMLE outperforms other methods in certain real-world settings, making it a useful and potentially superior algorithm for causal inference. However, there is a lack of accessible resources for practitioners to understand the implementation. Hence the TMLE framework is the least-used method by practitioners in epidemiology literature. Recently a few accessible articles have been published, but they focus only on binary outcomes and demonstrations are done mainly with simulated data. This paper aims to fill the gap in the literature by providing a step-by-step TMLE implementation guide for a continuous outcome, using an openly accessible clinical dataset.

## Introduction

The goal of causal inference is to extract the amount of change in the outcome caused exclusively by a change in the treatment of interest. This causal effect can be formally defined based on the counterfactual framework established by Rubin, discussed in detail later.^1^ In 2006, Van der Laan and Rubin developed targeted maximum likelihood estimation (TMLE),^2^ a promising causal inference method. TMLE is different from traditional methods in that it uses estimates of both the treatment-generating mechanism (propensity score model) and the outcome-generating mechanism to estimate the average treatment effect. The use of both of these models has a special advantage because TMLE is doubly robust, meaning it provides a consistent estimator if either the exposure or outcome model is correctly specified.^3^ TMLE has been shown to outperform inverse probability of treatment weighting (IPTW) and G-computation methods (discussed later), particularly when model misspecification is likely.^3–8^ Despite its gaining recognition in the statistical sciences, there is a shortage of resources accessible to practitioners who could benefit from using TMLE in real-world scenarios. In particular, though Luque-Fernandez et al. provide a well-written guide to TMLE for a binary outcome,^5^ such a tutorial for Gruber and van der Laan’s method for a continuous outcome has yet to be published.^6^ The steps are similar to those for a binary outcome, but there is little easily understandable high-level literature on the differences. Continuous outcomes are widespread in epidemiology, though they are often dichotomized for ease of analysis and to correlate directly with care decisions. The dichotomization of a continuous outcome, however, creates several problems. Firstly, it reduces the statistical power of the analysis since we are losing information about the outcome variable.^9^ Secondly, the calculated measures of association change with the cut point used to dichotomize the variable and the underlying distribution.^10^ Due to these risks, leaving the variable as a continuous outcome is often beneficial. The understanding of TMLE in the continuous outcome setting is therefore especially important. Additionally, previous guides on the implementation of TMLE have primarily focused on simulated data.^5,11^ This article aims to demonstrate the steps of TMLE to estimate the treatment effect using a real-world, openly accessible dataset to give practitioners a sense of how this method works in practice.^12^ We also explain the related approaches in an organized way, so that readers understand the difference between the commonly used methods and TMLE.

## Methods

### Rubin’s causal framework

Essentially, Rubin’s framework states that if in a study with a binary treatment A , some subjects are assigned the treatment (A=1) , and others are assigned a placebo or other comparative treatment (A=0) , we can only observe the outcome for each subject under the treatment they received. However, to assess the effect a treatment has on the outcome of interest, we need to compare the outcome in a particular subject under treatment with the outcome of that particular subject under no or other treatment. The counterfactual outcome is what the outcome for a certain subject would have been had they received a different level of treatment. Our goal, therefore, in determining the effect of a treatment on the outcome, is to estimate the counterfactual outcomes as accurately as possible. In observational studies, this is particularly challenging because the treated and untreated groups we are comparing often have very different covariate and confounder distributions. In other words, people with certain characteristics are often more likely to have received treatment than others, making it difficult to pinpoint the effect of the treatment.^13^

### Assumptions

To estimate the counterfactuals described in Rubin’s causal framework, and label the resulting treatment effect as causal, there are several assumptions we have to make.^14^

1. Conditional exchangeability: the conditional probability of receiving treatment depends only on the measured covariates. In other words, the unmeasured factors that affect the outcome are distributed equally between the treated and untreated groups, conditional on measured confounders.^13^
2. Positivity: for each combination of covariate levels, there must be a positive probability of each treatment condition (treated and untreated). The outcome of one subject may not depend on the treatment any other subject received.^13^
3. Consistency: the treatment must be well-defined so that there is no ambiguity about dosage, length of treatment, or other factors that could cause the level of exposure to differ between subjects. If we know the exposure is well-defined, we can assume a subject’s observed outcome will be equal to their corresponding counterfactual outcome given their observed exposure history.^15^

### G-computation method

Under the above assumptions, the average treatment effect (ATE) can be defined as the average outcome under treatment minus the average outcome under no treatment. Y(A=a) represents the outcome under treatment A=a . The average outcome under each treatment includes the observed outcomes as well as the counterfactual outcomes.

ATE=E[Y(A=1)]−E[Y(A=0)]

One of the methods commonly used to estimate the treatment effect using counterfactual outcomes is the G-computation method. It is based on modelling the outcome and uses linear regression models to predict the outcome for each subject under each treatment. All covariates, represented here as the matrix W in which each column constitutes the values of a particular covariate, are included in these models in order to account for confounding.^16^

$$E\left(Y|A,W\right)=\beta_{0}+\beta_{1}A+\beta_{2}^{T}W$$

For instance, for a binary treatment A , (i) we fit a linear model on the original data to obtain estimated coefficients associated with the parameters ( $\beta_{0},\beta_{1},\beta_{2}^{T}$ ). (ii) We then create a new data that is very similar to the original data (with respect to outcome and covariates), except the fact that treatment conditions for all subjects are set to treated (setting A=1 for all). (iii) This new data (with everyone assigned to be treated) is then used to predict the outcome Y for each subject based on the same estimated coefficients obtained from the fit from the original data. (iv) Similar to step (ii), we again create another new data that is very similar to the original data, except the fact that treatment conditions for all subjects are now set to untreated (setting A=0 for all). (v) This second new data (with everyone assigned to be untreated) is then used to predict the outcome Y for each subject based on the same estimated coefficients obtained from the fit from the original data. These predicted outcomes for each subject i from steps (iii) and (v) are our estimates of the counterfactual outcomes [ ${\hat{Y}}_{i}\left(a=1\right)$ and ${\hat{Y}}_{i}\left(a=0\right)$ respectively]. (vi) Our estimate of the average treatment effect is then simply the difference of the average predicted outcome under treatment A=1 and the average predicted outcome under treatment A=0 :^17,18^

$$\hat{ATE}=\frac{\overset{n}{\underset{i=1}{\sum }}\left[{\hat{Y}}_{i}\left(a=1\right)-{\hat{Y}}_{i}\left(a=0\right)\right]}{n}.$$

G-computation performs poorly under model misspecification and requires the use of bootstrap sampling, a tedious resampling technique, to calculate confidence intervals.^5^

### Inverse probability of treatment weighting (IPTW) method

Another common approach to estimate the treatment effect is the inverse probability of treatment weighting (IPTW), a propensity score-based estimation method that models the treatment-generating mechanism. The propensity score is the conditional probability of this observation being assigned the treatment given all measured covariates. The propensity score model, also called the treatment model, is typically estimated through logistic regression (shown for data with k covariates):

$$logit\left(A_{i}=1|W_{i}\right)=\beta_{0}+\beta_{1}W_{1i}+\ldots +\beta_{k}W_{ki}=\log\frac{P\left(A_{i}=1|W_{i}\right)}{1-P\left(A_{i}=1|W_{i}\right)}.$$

The propensity scores are then:

$$g_{i}\left(W_{i}\right)=P\left(A_{i}=1|W_{i}\right)=\frac{\exp\left(logit\left(A_{i}=1|W_{i}\right)\right)}{1+\exp\left(logit\left(A_{i}=1|W_{i}\right)\right)}.$$

The weights for the average treatment effect (ATE) are calculated as follows:

$$w_{i}=\frac{A_{i}}{g_{i}\left(W_{i}\right)}+\frac{1-A_{i}}{1-g_{i}\left(W_{i}\right)}.$$

Those who were very likely to receive the treatment and did receive the treatment will be weighted less heavily than the treated who were unlikely to receive treatment, artificially balancing the distribution of covariates between the two groups.^19^ The weights for each observation are multiplied by the corresponding outcome. When a particular observation is treated ( $A_{i}=1$ ), the second part of the weight function falls away, leaving the first term. The opposite is true when the observation is untreated ( $A_{i}=0)$ . The average treatment effect is then calculated as the difference between the average weighted outcome of the treated and the average weighted outcome of the untreated.

$$\hat{ATE}=\frac{1}{n}\overset{n}{\underset{i}{\sum }}\left[\frac{A_{i}Y_{i}}{\hat{g}\left(W_{i}\right)}-\frac{\left({1-A}_{i}\right)Y_{i}}{1-\hat{g}\left(W_{i}\right)}\right].$$

IPTW estimates can be unstable with a high variance if there are propensity score values close to 0, leading to large weights. The method is also sensitive to model misspecification, especially with a large number of variables.^5^

### TMLE

Targeted maximum likelihood estimation (TMLE) combines an outcome model based on the G-computation framework with an exposure model based on IPTW to estimate the parameter of interest.^20^ Incorporating both models makes this method doubly robust, so the estimate of our parameter of interest will be consistent if either the outcome or exposure model is correctly specified. A consistent estimator essentially means the estimate gets closer and closer to the true value as the sample size increases.^11^ If both models are correctly specified, the estimate will be efficient, meaning a smaller sample size is required for a narrow confidence interval than if one method were misspecified.^3^ A further benefit of TMLE is that it allows for the use of machine learning algorithms in generating both the treatment (propensity score) and outcome models. This is not possible in the pure propensity score and G-computation methods because the sampling distribution of the estimate resulting from the use of machine learning to directly estimate the treatment effect generally does not approximate a known distribution.^21^ Usually, a standard regression using maximum likelihood estimation will give an estimate that follows an approximate normal distribution, assuming the model was correctly specified. Statistical inference such as standard errors and confidence intervals are then straightforward to calculate. By contrast, if machine learning is used directly in a model from which the estimate is extracted directly, statistical inference is not straightforward since the estimator does not follow a known distribution.^21^ TMLE, on the other hand, uses machine learning only in intermediary steps, rather than to estimate the parameter of interest directly. The sampling distribution of our estimate is therefore approximately normal and statistical inference is still straightforward. Data-adaptive modelling allows TMLE to require more relaxed assumptions about the data structure than other methods, aiding in the minimization of bias and the construction of an efficient estimator.^3^ It has been shown that doubly robust estimators using machine learning, such as TMLE, outperform singly robust estimators using machine learning, such as IPTW.^22^

For a binary outcome, as shown in previous tutorials,^5^ the process of implementing TMLE is fairly straightforward. Since the range of a binary outcome is completely predictable, it is guaranteed that all our estimated outcomes will be inside the 0-1 range. If the same approach is used for a continuous outcome, the assumption that the modeled outcomes lie within the range of the true distribution may not hold.^6^ Predicted outcomes outside the true distribution’s range can result in increased bias and variance in the estimator, particularly in sparse data settings.^3,6^ In 2010, Susan Gruber and Mark J. van der Laan suggested a slightly modified approach to TMLE with a bounded continuous outcome.^6^ They suggest the same approach to be used for continuous outcomes where the bounds are not known a priori by setting the minimum and maximum of the sample outcome as the bounds. This approach uses a logistic fluctuation function rather than the linear function typically used for continuous outcomes.

### SuperLearner

In this tutorial we apply SuperLearner as the machine learning algorithm to generate the treatment and outcome models.^23^ SuperLearner is an ensemble machine learning technique that can either (i) select from or (ii) combine different machine learning techniques into one “super learner” to make outcome predictions based on a set of given covariates. In the selection version, SuperLearner uses cross-validation to select the best machine learning algorithm for the given dataset from a list of candidate algorithms provided by the user. The combination version uses cross-validation to select the best possible linear combination of the candidate algorithms provided.^23^ The *SuperLearner* package in R implements versions of both, with a default list of candidate machine learning algorithms.^24^ SuperLearner will always be at least as accurate as the best algorithm included in the set of algorithms it can draw from.^24^ It is, therefore, the recommended approach for constructing both the treatment and outcome models in TMLE.^3^

### Motivating example: Right heart catheterization

This guide will go through a step-by-step example using the right heart catheterization (RHC) dataset. This dataset of 5735 adult patients was used by Connors et al. in their 1996 study of the effectiveness of RHC in the initial care of critically ill patients.^12^ Included patients were receiving care in an intensive care unit (ICU) for one of nine prespecified disease categories and the group was selected to have a total 6-months mortality of 50%. The original study used propensity score matching to analyze the effect of receiving RHC in the first 24 h of care in the ICU on survival time, cost of care, intensity of care, and length of stay in the ICU and hospital. The variables to include in the propensity score were chosen with the expertise of a panel of four intensivists and three cardiologists. In this example, we focus on the continuous outcome of length of stay. The study by Connors et al. reported an increase of 1.8 days in length of stay for patients who received RHC compared to those who did not. The code we used to prepare the dataset can be found in Box 14 in the supplementary materials.

### TMLE steps

We perform steps 2–7 (Table 1) to obtain point estimates when dealing with a binary outcome.^5^ For our setting, with a continuous outcome, we need to add steps 1 and 8 for the transformation of the outcome. These steps make up two general stages of TMLE. The first stage consists of steps 1 and 2 and represents the initial construction of the outcome model and the first crude estimate of the treatment effect. The second stage is the targeting stage of TMLE, consisting of steps 3 to 8. The targeting step aims to refine our initial estimate in the direction of the true value of the parameter we are interested in.

**Table 1.** Targeted maximum likelihood estimation (TMLE) steps when the outcome of interest is continuous.

Step #	Description of the TMLE steps
1	Transformation of the continuous outcome variable
2	Predict from initial outcome modelling: G-computation
3	Predict from propensity score model
4	Estimate clever covariate H
5	Estimate fluctuation parameter ϵ
6	Update the initial outcome model prediction based on targeted adjustment of the initial predictions using the PS model
7	Find treatment effect estimate
8	Transform back the treatment effect estimate to the original (continuous) outcome scale
9	Confidence interval estimation based on closed-form formula

## Step-by-Step Analysis

### Step 1: Transformation of the continuous outcome variable

With a binary outcome, our initisal crude estimate of the treatment effect Ψ would simply be the difference between the average observed exposed and unexposed outcomes given covariates W :

$$\Psi \left(P_{0}\right)=E_{0}\left(E_{0}\left(Y|A=1,W\right)-E_{0}\left(Y|A=0,W\right)\right).$$

A key step in TMLE for a continuous outcome is to transform our outcome Y to be within a range of 0–1, which enables us to use a logistic regression to calculate our initial estimate.^3^ If we were to use a linear regression, some predictions could fall outside the range we assume for our data model. Say our Y is within the range [a,b] . To transform our outcome, we can use min-max normalization, letting:^3,25^

$$Y^{*}=\frac{Y-a}{b-a}.$$

The R code for the transformation of Y is shown in Box 1. Our parameter of interest becomes:

$$\Psi^{*}\left(P_{0}\right)=E_{0}\left(E_{0}\left(Y^{*}|A=1,W\right)-E_{0}\left(Y^{*}|A=0,W\right)\right).$$

The parameter of interest on the transformed scale relates to our original parameter of interest Ψ by simple multiplication:

$$\Psi \left(P_{0}\right)=\left(b-a\right)\Psi^{*}\left(P_{0}\right).$$

If our outcome is technically unbounded, we can use the minimum and maximum outcomes found in our sample data as a and b , or set an upper bound for our data and perform the same transformation.^3^

## Box 1 – Transforming the outcome Y to the scale [0,1]


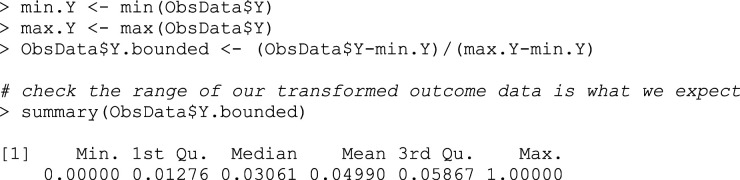


### Step 2: Predict from initial outcome modelling: G-computation

In the first stage of TMLE, we need to calculate our starting point. This means following the G-computation framework to find an initial crude estimate of the ATE. The use of data-adaptive methods such as SuperLearner is recommended for this step, since they are less likely to result in model misspecification than parametric methods since they make no a priori assumptions about the data model. In TMLE, unlike singly robust methods such as propensity score methods, the use of machine learning algorithms does not come at the cost of interpretability.^3^

Using the *SuperLearner* package, we can fit an initial model for the outcome Y to our dataset, and use the fitted model to make our initial predictions (Box 2). An important note here is that rather than using a linear loss function as we would typically do when performing maximum likelihood estimation (MLE) with a continuous outcome, it is recommended that we use a logistic loss function.^6^ Using a linear loss function would create a model that would allow predicted values to fall outside the range originally seen in our data.^3^ In sparse data settings, this can cause increased bias and variance. The use of a logistic loss function, on the other hand, has been shown to be at least as good as the linear loss function approach, and better in sparse data scenarios. This loss function takes the following form, in which Y represents the observed outcome and $\bar{Q}\left(A,W\right)$ represents the predicted outcome given covariates W and exposure A ^6^:

$$-L\left(\bar{Q}\right)=Y\log\left(\bar{Q}\left(A,W\right)\right)+\left(1-Y\right)\log\left(1-\bar{Q}\left(A,W\right)\right).$$

In the context of SuperLearner, this means we set the *method* parameter to be “method.CC_nloglik” which lets the algorithm know we want to use the log-likelihood loss function.^24^

## Box 2 – Fitting the initial outcome model using SuperLearner and making predictions for observed treatment assignments


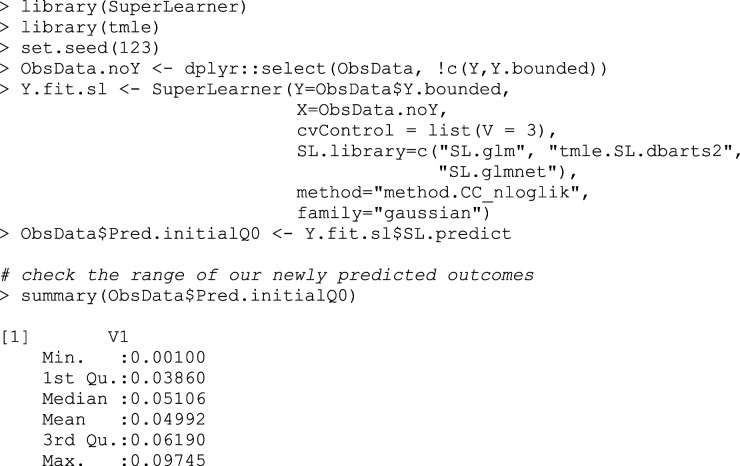


As well as predicting the outcome under treatment received, we also predict the outcomes for each subject under each theoretical treatment, regardless of what treatment they truly received (Box 3a,3b).

## Box 3a – Making predictions ${\bar{Q}}^{0}\left(1,W\right)$ for the counterfactual outcome, assigning all treatments to A = 1


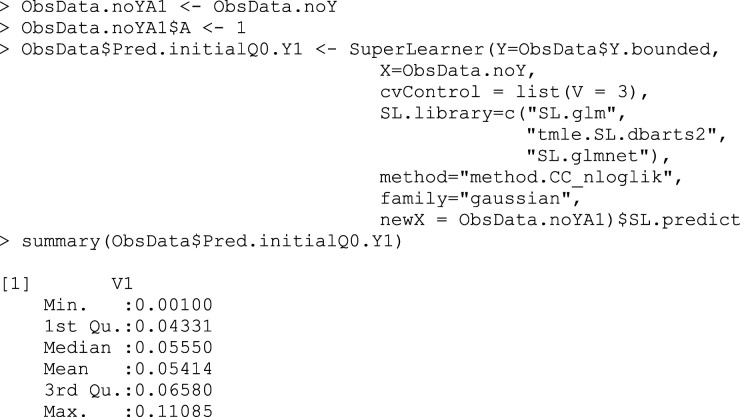


## Box 3b – Making predictions ${\bar{Q}}^{0}\left(0,W\right)$ for the counterfactual outcome, assigning all treatments to A = 0


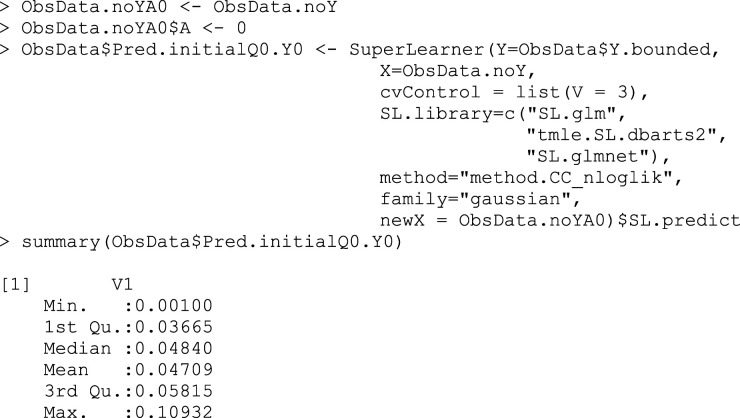


These values, ${\bar{Q}}^{0}\left(0,W\right)$ and ${\bar{Q}}^{0}\left(1,W\right)$ , represent an estimate of the full set of counterfactual outcomes we would have in an ideal world.^11^ ${\bar{Q}}^{0}\left(0,W\right)$ represents the predicted outcome of a subject had they received an RHC given covariates W , and ${\bar{Q}}^{0}\left(1,W\right)$ represents the predicted outcome had they not received an RHC, given the same covariates W . The average difference of these values gives us our initial estimate of the treatment effect (Box 4).

## Box 4 – Calculating the crude ATE estimate using counterfactual outcome predictions


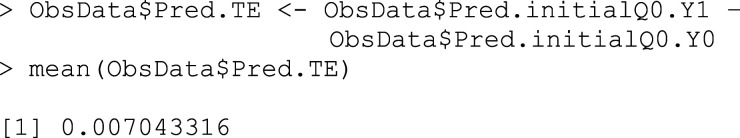


### Step 3: Predict from propensity score model

The next step is to construct the propensity score model to be used in our calculation of the so-called clever covariates and a fluctuation parameter ϵ (Box 5). The typical propensity score model based on logistic regression would have the form^19^:

$$\hat{g}\left(1,W\right)=\frac{\exp\hat{\alpha_{0}}+\hat{\alpha_{1}^{T}}W}{1+\exp\hat{\alpha_{0}}+\hat{\alpha_{1}^{T}}W}.$$

However, it is again recommended to use a SuperLearner to construct the propensity score model, then use the fitted model to predict the probabilities $P\left(A|W\right)=\hat{g}\left(1,W\right)$ for each set of covariate values W .^3^

## Box 5 – Constructing the propensity score models and extracting the propensity scores through prediction


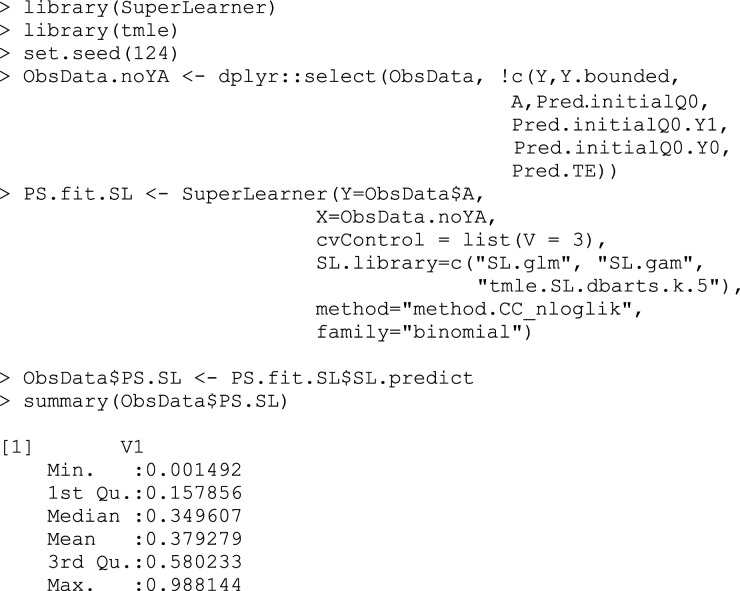


### Step 4: Estimate clever covariate H

To update our estimate, we define the parametric submodel:

$$Y=\epsilon H\left(A,W\right)+offset\left({\bar{Q}}_{n}^{0}\right)$$

We set ${\bar{Q}}_{n}^{0}$ to be the model’s offset, so that ϵH(A,W) gives the difference between our initial estimate and the true outcome. The offset fixes the coefficient of the initial estimate ${\bar{Q}}_{n}^{0}$ at 1 so the model does not estimate a parameter associated with the initial estimate.^21^ H(A,W) is a “clever covariate” that we estimate for each individual, based on their propensity score. It consists of two parts, $H_{0}\left(A,W\right)$ and $H_{1}\left(A,W\right)$ .^3^ For individual i , for example:

$H_{0}\left(A_{i},W_{i}\right)=\left(\frac{I\left(A_{i}=0\right)}{g\left(A_{i}=0|W_{i}\right)}\right)$ and $H_{1}\left(A_{i},W_{i}\right)=\left(\frac{I\left(A_{i}=1\right)}{g\left(A_{i}=1|W_{i}\right)}\right)$ where g(A,W) is the propensity score model (Box 6). Note that the two components of the clever covariate look similar to the weights used in IPTW.

The clever covariate H(A,W) is derived from the influence curve. Our data has a certain sample distribution, likely not exactly equal to the true distribution of the population from which we drew our sample. An influence curve describes how an estimator (for instance, the average treatment effect) changes when the data distribution varies slightly.^5^ In other words, it describes the way sampling variation affects the estimator of interest. An efficient influence curve is the influence curve with the least variance for a particular estimator.^5^

## Box 6 – Using the propensity scores to calculate the clever covariates


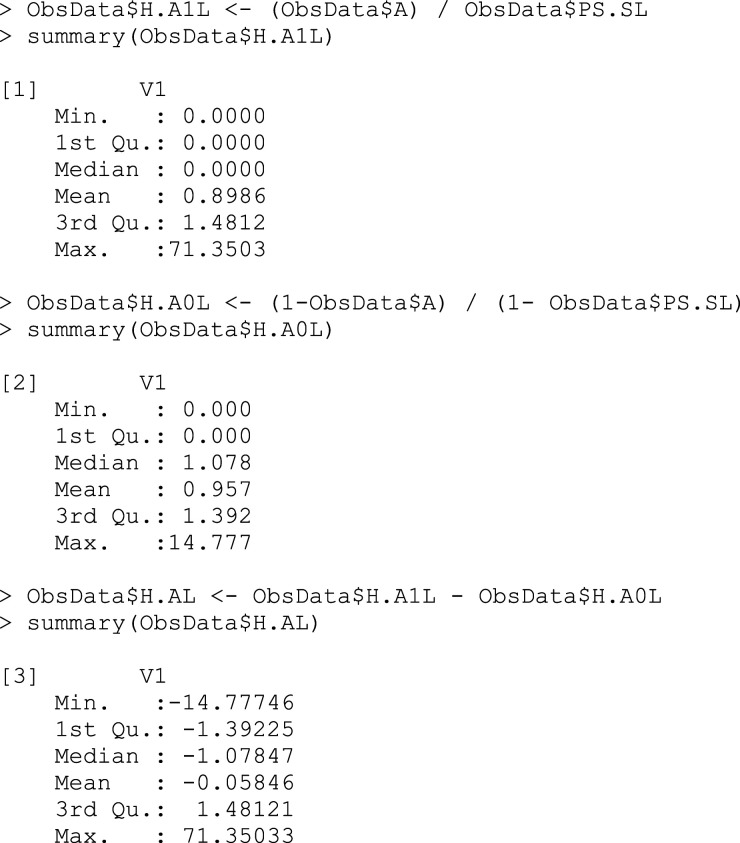


The clever covariate allows us to target the update of our estimate towards the true value of the parameter we are interested in. The weights are higher for the cases with an unlikely treatment-covariate combination, since they are underrepresented in the dataset compared to the ideal scenario in which the groups would be completely comparable. The weighted treated and untreated observations will thus be artificially balanced in terms of all the other covariates.

### Step 5: Estimate fluctuation parameter ϵ

We also need to estimate our fluctuation parameter. The fluctuation parameter $\hat{\epsilon }=\left(\hat{\epsilon_{0}},\hat{\epsilon_{1}}\right)$ is estimated through maximum likelihood estimation, with a model in which the logit of the initial estimate ( $\bar{Q^{0}}\left(A,W\right)$ ) is set as an offset, and the clever covariates are used as independent variables as shown in Box 7:^11^

$$E\left(Y=1|A,W\right)\left(\epsilon \right)=\frac{1}{1+\exp\left(-\log\frac{\bar{Q^{0}}\left(A,W\right)}{\left(1-\bar{Q^{0}}\left(A,W\right)\right)}-\epsilon_{0}H\left(0,W\right)-\epsilon_{1}H\left(1,W\right)\right)}.$$

When the initial estimate of $\bar{Q^{0}}\left(A,W\right)$ is close to the true value of the outcome, the fluctuation parameter ϵ will be close to 0. In other words, there will be little adjustment made to the initial estimate. This is the case, for example, when the initial estimate was correctly specified and there was no residual confounding. ϵ will also be close to 0 if the exposure model does not provide any more information relevant to the outcome Y .^5^

## Box 7 – Using the clever covariates and the initial predictions to estimate the fluctuation parameter ϵ


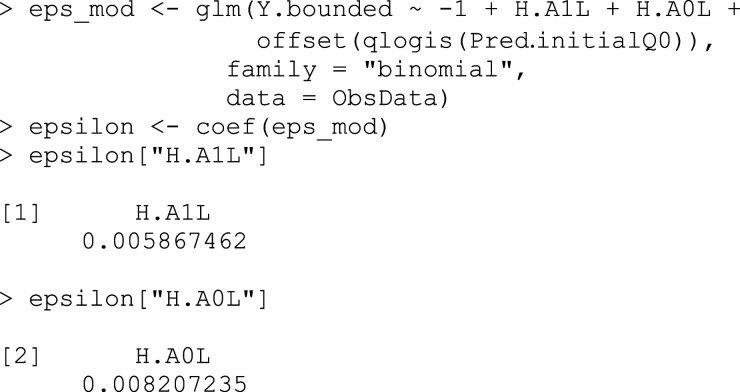


An alternative approach, which we will not demonstrate here, is to use clever covariates as weights rather than covariates in this step, which can improve the stability of the estimate.^5^

### Step 6: Update the initial outcome model prediction based on targeted adjustment of the initial predictions using the PS model

For this step, we define the expit function as follows:

$$expit\left(x\right)=\frac{\exp\left(x\right)}{1+\exp\left(x\right)}$$

The update to our initial estimate is performed by plugging in the ϵ calculated in the previous step into the following equations for all subjects (Box 8). This effectively nudges our initial estimate in the direction of the true average outcome.^5^

$$\bar{Q^{1}}\left(0,W\right)=expit\left[logit\left(\bar{Q^{0}}\left(0,W\right)\right)+\hat{\epsilon_{0}}H\left(0,W\right)\right].$$

$$\bar{Q^{1}}\left(1,W\right)=expit\left[logit\left(\bar{Q^{0}}\left(1,W\right)\right)+\hat{\epsilon_{1}}H\left(1,W\right)\right].$$

## Box 8 – Updating predicted counterfactual outcomes using the clever covariates and fluctuation parameter


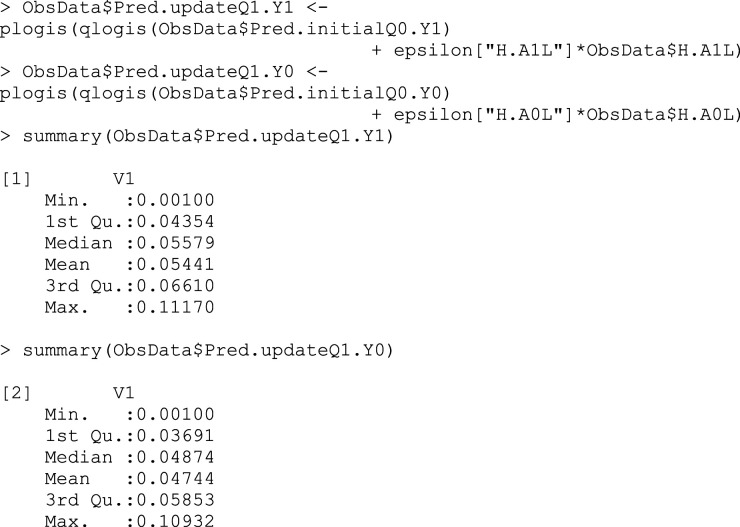


### Step 7: Find treatment effect estimate

The targeted estimate of the ATE is calculated using the following formula, which is an average of the difference between the targeted estimates of the outcomes when treated versus not treated (Box 9)^5^:

$${\hat{ATE}}_{TMLE}^{*}=\frac{1}{n}\overset{n}{\underset{i=1}{\sum }}\bar{Q^{1}}\left(1,W_{i}\right)-\bar{Q^{1}}\left(0,W_{i}\right).$$

## Box 9 – Calculating the ATE by the average difference between updated treated and untreated counterfactual outcomes


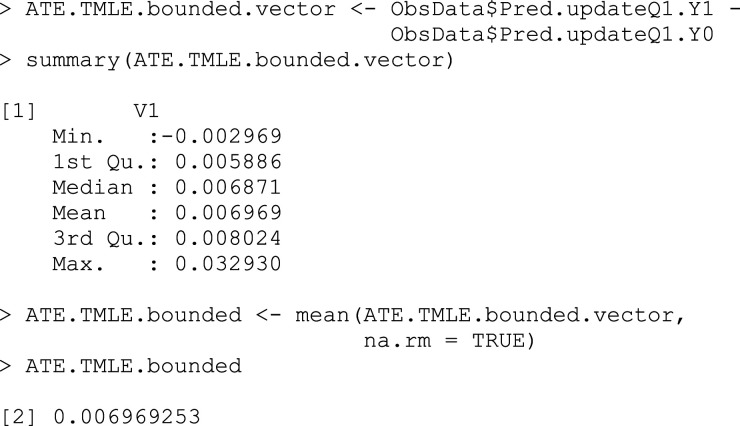


### Step 8: Transform back the treatment effect estimate to the original outcome scale

Note that the estimate from the previous step is still on the transformed outcome scale. To interpret our estimate on the original scale, we must use the original bounds of our outcome to transform this estimate back, as shown in Box 10.^3^

$${\hat{ATE}}_{TMLE}^{*}=\left(b-a\right){\hat{ATE}}_{TMLE}^{*}.$$

## Box 10 – Transforming the ATE back to the original outcome scale


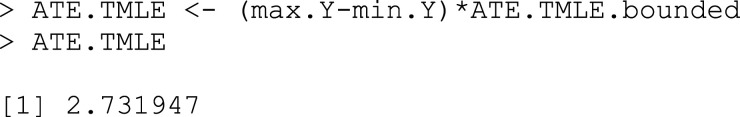


### Step 9: Confidence interval estimation

As always, we would like to calculate confidence intervals for our estimated ATE. To do this, we again use the influence function. The influence function has a mean of 0 and a finite variance, which, when divided by the number of observations, corresponds to the variance of the target parameter. There are many influence function options, but there is always an *efficient* influence function that achieves the lower bound on asymptotic variance (under the modeling assumptions). By finding this efficient influence function and calculating its variance, we can estimate the standard deviation of the parameter and construct confidence intervals for our parameter estimate (Box 11).^5^ For the ATE, the efficient influence function is:

$$EIC_{ATE}=\left(\frac{A}{P\left(A=1|W\right)}-\frac{1-A}{P\left(A=0|W\right)}\right)\left[Y-E\left(Y|A,W\right)\right]+E\left(Y|A=1,W\right)-E\left(Y|A=0,W\right)-ATE.$$

We can estimate this for each subject as:

$${\hat{EIC}}_{ATE}=\left(\frac{A}{\hat{g}\left(1,W\right)}-\frac{1-A}{\hat{g}\left(0,W\right)}\right)\left[Y-\bar{Q^{1}}\left(A,W\right)\right]+\bar{Q^{1}}\left(1,W\right)-\bar{Q^{1}}\left(0,W\right)-{\hat{ATE}}_{TMLE}.$$

The standard error estimate for our ATE estimate is then:

$${\hat{\sigma }}_{ATE,TMLE}=\sqrt{\frac{\hat{Var}\left({\hat{EIC}}_{ATE}\right)}{n}}.$$

## Box 11 – Confidence interval estimation using the efficient influence curve


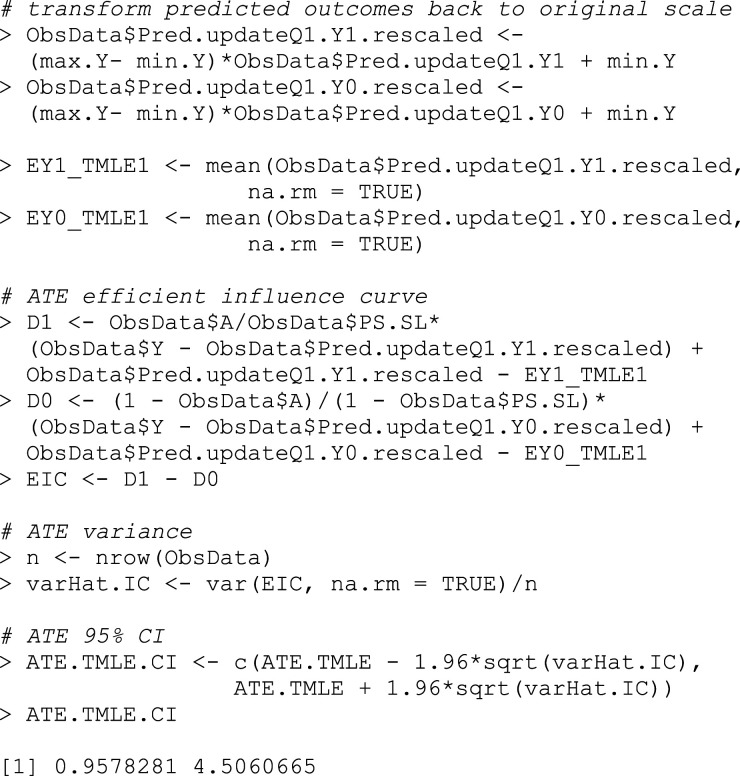


## Pre-packaged TMLE Software

To provide a clear explanation of how TMLE works, we have shown the implementation of each separate step so far. Once the readers understand the implementation details, they can also take advantage of several software packages in R that implement the TMLE steps.^26^

### tmle

The *tmle* package can handle both binary and continuous outcomes, and uses SuperLearner to construct the exposure and outcome models just like we did in the steps above (Box 12). Note also that the outcome Y is required to be within the range of [0,1] , so we need to pass in the transformed data to the *tmle* function, then transform the result back to the original scale.

The package’s (version 1.5.0.2) default SuperLearner library for estimating the outcome includes generalized linear models (GLMs), GLM with elastic net regularization, and Bayesian additive regression trees. The default library for estimating the propensity scores also includes GLMs and Bayesian additive regression trees (though specified slightly differently), and replaces the GLM with regularization with generalized additive models (GAMs).^27^ More methods can be added by specifying lists of models in the *Q.SL.library* (for the outcome model) and *g.SL.library* (for the propensity score model) arguments.

## Box 12 – Estimating the ATE using the tmle package


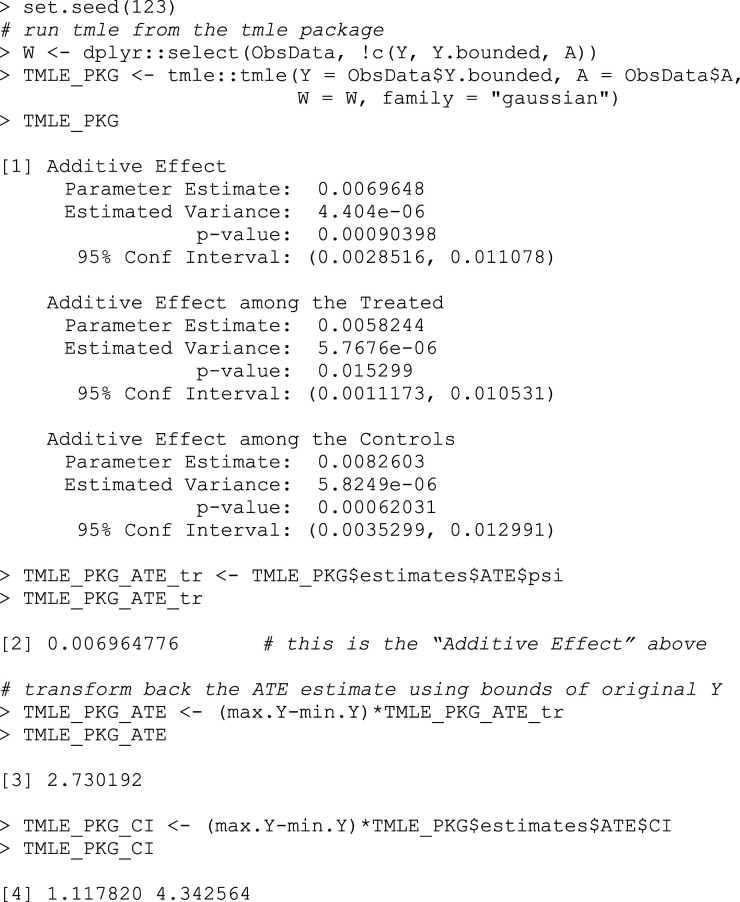


 In our first run-through of the step-by-step TMLE process, we used the SuperLearner libraries that the *tmle* package uses as its default. Our results show a difference in length of stay of 2.73 (0.96, 4.51) days between those who received RHC and those who did not. The point estimate is very similar to the 2.73 (1.12, 4.34) days difference returned by the *tmle* package. Slight differences could be attributed to the fact that there is some randomness always associated with machine learning algorithms, as you can run the same algorithm multiple times on the same dataset and get slightly different results. Overall, this supports that the steps given in this tutorial are a valid implementation of TMLE and can be referred to for an understanding of how the *tmle* package works.

## Other results reported in literature

### Keele paper

Luke Keele and Dylan Small studied a comparison of matching methods and machine learning methods including TMLE in a 2021 study.^28^ They reassessed several different datasets, including the RHC dataset with the length of stay outcome. To compare this study’s results with the results of our step-by-step guide, we reran the above steps with the same library of algorithms Keele et al. used in their assessment (Box 13, supplementary materials). The set of algorithms was the same for both the outcome and propensity score models and included generalized linear models (logistic regression), least absolute shrinkage and selection operator (lasso), and random forests. Keele and Small found an average difference in length of stay of 2.01 (0.6, 3.41) days with TMLE with SuperLearner.^28^ Our results with the same set of candidate machine learning algorithms showed a difference in length of stay of 2.44 (1.68, 3.20) days. This is not a small difference in results, both in terms of the point estimate and the confidence intervals. Since we did not have sufficient information to replicate the results exactly, it is possible that the difference in results could be attributed to different combinations of variables included in each analysis. The other possibility is that the differences were caused by the random sampling associated with cross-validation in SuperLearner. To minimize the effects of random sampling, we recommend following the flowchart given by Phillips et al. (2022) to select the number of cross-validation folds appropriate for the given sample size.^29^

### Original connors paper

The original study reported a difference in length of stay of 1.8 days between those treated with an RHC and those not treated with an RHC. This analysis was performed using propensity score matching, a method using the propensity scores calculated in IPTW as a variable to match on rather than as weights on individual observations.^12^ The result reported by Connors et al. is not very different from the result reported by Keele and Small, but all TMLE implementations examined resulted in a greater difference than that reported in the initial study, suggesting that the propensity score method’s estimate is probably an underestimate. Another study by Samuels et al. (2018) also used TMLE, among other methods, to reassess this dataset with the length of stay outcome. They report an ATE of 2.73 (1.22, 4.23) for the TMLE method with SuperLearner (implemented using the *tmle* package, with the default SuperLearner library).^30^ This result is again very close to the estimate we got from our TMLE implementation, likely because we used the same library of methods for our SuperLearner that is used by default in the TMLE package.^26^ The same study also compared other methods such as bagged one-to-one matching. The estimates using these methods were also higher than the 1.8 days estimated by Connors et al. in the original study. Though we cannot know the true treatment effect, this agreement between TMLE and other methods further supports that the effect reported in the initial study could have been underestimated.

## Discussion

In this article, we have described a step-by-step process for implementing TMLE in a continuous outcome setting. This application of TMLE to a well-known real-world dataset should provide practitioners with a transparent understanding of how this method can be used in similar epidemiological settings. Though overall the implementation is similar to the steps used for a binary treatment, it is crucial that we use a transformed outcome and specify a log-likelihood loss function in order to keep the desirable properties TMLE was designed to exhibit.^6^ This tutorial complements Luque-Fernandez’s tutorial on TMLE with a binary outcome.^5^ Through step-by-step instructions with complete code for an openly accessible dataset, we provide an easy-to-follow and reproducible guide for the application of TMLE in a real-world scenario with a common outcome format. We highlight the benefits of using TMLE with machine learning and validate the results from this guide with those found in literature for the same dataset.

The benefits of using TMLE include the fact that it is doubly robust and thus consistent if either the propensity score model or the outcome model is correctly specified. Additionally, the possibility of using machine learning methods to construct each model decreases the likelihood that the models will be misspecified, while still allowing valid statistical inference with 95% confidence intervals. When using machine learning methods such as SuperLearner in TMLE, it is important to select good parameters for the data in question, including an adequate number of cross-validation folds and a reasonable library of candidate learners. To select appropriate parameters for a certain analysis, we recommend following the guidelines given by Phillips et al. (2022) and Balzer et al. (2021).^29,31^ TMLE can also be adapted to work with a variety of different outcomes such as longitudinal outcomes,^32^ and even different types of treatments such as multiple time point interventions.^33^ Recently, TMLE has also been adapted to settings with dependent exposures^34^ and time-to-event outcomes,^35^ TMLE for use with decision trees and regional exposures,^36^ as well as a higher order TMLE that may have improved performance.^37^

Augmented inverse propensity weighting (AIPW) is another doubly robust method available. AIPW uses information from the outcome regression model to tweak the inverse probability weighted estimator.^13^ The augmentation term based on the outcome model helps to correct the estimate when the treatment mechanism was misspecified.^38^ Despite this, TMLE has been shown to provide less biased estimates than AIPW since TMLE is more robust to near positivity violations and TMLE is a plug-in estimator restricting probabilities to be between 0 and 1.^13^ Recently, an R package specifically for AIPW estimation was made available.^39^

Despite its usefulness, TMLE is not perfect. It still relies on the assumption that there is no unmeasured confounding. Often there are variables we cannot measure or do not even know about, that may influence the exposure and outcome. Unmeasured confounding leads to model misspecification, so TMLE’s assumption that at least one of the outcome and exposure models is correctly specified may not hold. In this case, the TMLE estimator will not be consistent and we may need to use a different method. Additionally, TMLE does not differentiate between instruments and true confounders in the propensity score model, but includes either as long as there is a relationship between the variable and the exposure.^3^ This can lead to near or full positivity violations because when we use a SuperLearner to construct the exposure model, it may almost perfectly predict the exposure given a covariate strongly related to the exposure.^40^ Improvements could be made by using a double cross-fit procedure with TMLE. This method, by fitting the outcome and treatment models in each split of the data and predicting outcomes using models from discordant splits, addresses non-linearity bias.^41^

Future tutorials could focus on different outcome or treatment types, or variations of TMLE.^21^ For instance, Collaborative TMLE (CTMLE) is an advancement of TMLE that uses multiple iterations of TMLE to generate an estimate that under certain conditions is consistent even if both models are misspecified.^42^ Other extensions could include longitudinal data or multiple time point interventions or other types of data often encountered by practitioners in the real world but complex to analyze.

## References

[1] Rubin DB . Estimating causal effects of treatments in randomized and nonrandomized studies. J Educ Psychol 1974; 66(5): 688–701.

[2] Laan MJ Rubin D . Targeted maximum likelihood learning. U.C. Berkeley division of biostatistics working paper series. Berkeley, CA: Bepress, 2006. http://biostats.bepress.com/ucbbiostat/paper213.

[3] Van Der Laan MJ Rose S . Targeted learning: causal inference for observational and experimental data. Berlin, Germany: Springer, 2011.

[4] Pang M Schuster T Filion KB , et al. Targeted maximum likelihood estimation for pharmacoepidemiologic research. Epidemiology 2016; 27(4): 570–577. 27031037 10.1097/EDE.0000000000000487 PMC4890840

[5] Luque-Fernandez MA Schomaker M Rachet B , et al. Targeted maximum likelihood estimation for a binary treatment: a tutorial. Stat Med 2018; 37: 2530–2546. 29687470 10.1002/sim.7628 PMC6032875

[6] Gruber S Van Der Laan MJ . A targeted maximum likelihood estimator of a causal effect on a bounded continuous outcome. Int J Biostat 2010; 6(1): 26. 21731529 10.2202/1557-4679.1260 PMC3126669

[7] Ren J Cislo P Cappelleri JC , et al. Comparing g-computation, propensity score-based weighting, and targeted maximum likelihood estimation for analyzing externally controlled trials with both measured and unmeasured confounders: a simulation study. BMC Med Res Methodol 2023; 23(1): 11–18. 36647031 10.1186/s12874-023-01835-6 PMC9843888

[8] Amusa L Zewotir T North D . The impact of unmeasured confounding on causal inference in observational studies: a plasmode simulation study of targeted maximum likelihood estimation. Songklanakarin J Sci Technol 2022; 44(2): 474–480.

[9] Selvin S . Two issues concerning the analysis of grouped data. Eur J Epidemiol 1987; 3(3): 284–287. 3653356 10.1007/BF00149737

[10] Ragland DR . Dichotomizing continuous outcome variables: dependence of the magnitude of association and statistical power on the cutpoint. Epidemiology 1992; 3(5): 434–440. 1391136 10.1097/00001648-199209000-00009

[11] Gruber S Laan MJ . Targeted maximum likelihood estimation: a gentle introduction. U.C. Berkeley division of biostatistics working paper series. Berkeley, CA: Bepress, 2009. Report No.: 252.

[12] Connors AF Speroff T Dawson N V. , et al. The effectiveness of right heart catheterization in the initial care of critically ill patients. SUPPORT Investigators. J Am Med Assoc 1996; 276(11): 889–897. 10.1001/jama.276.11.889 8782638

[13] Smith MJ Mansournia MA Maringe C , et al. Introduction to computational causal inference using reproducible Stata, R, and Python code: a tutorial. Stat Med 2022; 41(2): 407–432. 34713468 10.1002/sim.9234 PMC11795351

[14] Chatton A BorgneLe F Leyrat C , et al. G-computation, propensity score-based methods, and targeted maximum likelihood estimator for causal inference with different covariates sets: a comparative simulation study. Scientific Reports 2020; 10: 1–13. 32514028 10.1038/s41598-020-65917-x PMC7280276

[15] Vander Weele TJ . Concerning the consistency assumption in causal inference. Epidemiology 2009; 20(6): 880–883. 19829187 10.1097/EDE.0b013e3181bd5638

[16] Snowden JM Rose S Mortimer KM . Implementation of G-computation on a simulated data set: demonstration of a causal inference technique. Am J Epidemiol 2011; 173(7): 731–738. 21415029 10.1093/aje/kwq472 PMC3105284

[17] Chatton A Le Borgne F Leyrat C , et al. G-computation, propensity score-based methods, and targeted maximum likelihood estimator for causal inference with different covariates sets: a comparative simulation study. Sci Rep 2020; 10: 9219. 32514028 10.1038/s41598-020-65917-x PMC7280276

[18] Le Borgne F Chatton A Léger M , et al. G-computation and machine learning for estimating the causal effects of binary exposure statuses on binary outcomes. Sci Rep 2021; 11: 1435. 33446866 10.1038/s41598-021-81110-0 PMC7809122

[19] Leite W . Practical propensity score methods using R. Thousand Oaks, CA: Sage Publications, Inc, 2017.

[20] Schuler MS Rose S . Targeted maximum likelihood estimation for causal inference in observational studies. Am J Epidemiol 2017; 185(1): 65–73. 27941068 10.1093/aje/kww165

[21] van der Laan MJ Rose S . Targeted learning in data science: causal inference for complex longitudinal studies. Berlin, Germany: Springer, 2018, p. 640.

[22] Naimi AI Mishler AE Kennedy EH . Challenges in obtaining valid causal effect estimates with machine learning algorithms. Am J Epidemiol 2021; kwab201, doi:10.1093/aje/kwab201. 34268558 PMC12096307

[23] Van Der Laan MJ Polley EC Hubbard AE . Super learner. Stat Appl Genet Mol Biol 2007; 6(1): 25. 10.2202/1544-6115.1309 17910531

[24] Polley EC LeDell E Kennedy C , et al. SuperLearner: super learner prediction, 2021. https://github.com/ecpolley/SuperLearner.

[25] Kiran A Vasumathi D . Data mining: min–max normalization based data perturbation technique for privacy preservation. In: Proceedings of the third international conference on compuational intelligence and informatics. Singapore: Springer, 2020, pp. 723–734.

[26] Gruber S Laan MJ . tmle: an R package for targeted maximum likelihood estimation. J Stat Softw 2012; 51(13).

[27] Gruber S van der Laan M Kennedy C . Tmle: targeted maximum likelihood estimation, 2021. https://cran.r-project.org/package=tmle.

[28] Keele L Small DS . Comparing covariate prioritization via matching to machine learning methods for causal inference using five empirical applications. Am Stat 2021; 75(2): 355–363.

[29] Phillips R V Van Der Laan MJ Lee H , et al. Practical considerations for specifying a super learner. Int J Epidemiol 2023; dyad023, doi:10.1093/ije/dyad023. 36905602

[30] Samuels LR Greevy RA . Bagged one-to-one matching for efficient and robust treatment effect estimation. Stat Med 2018; 37: 4353–4373. 30101483 10.1002/sim.7926

[31] Balzer LB Westling T . Demystifying statistical inference when using machine learning in causal research. Am J Epidemiol 2021; kwab200, doi:10.1093/aje/kwab200. 34268553 PMC10472326

[32] Petersen ML Schwab J Gruber S , et al. Targeted maximum likelihood estimation for dynamic and static longitudinal marginal structural working models. J Causal Inference 2014; 2(2): 147–185. 25909047 10.1515/jci-2013-0007 PMC4405134

[33] Van Der Laan MJ Gruber S . Targeted minimum loss based estimation of causal effects of multiple time point interventions. Int J Biostat 2012; 8(1). 10.1515/1557-4679.1370 22611591

[34] Zivich PN Hudgens MG Brookhart MA , et al. Targeted maximum likelihood estimation of causal effects with interference: a simulation study. Stat Med 2022; 41(23): 4554–4577. 35852017 10.1002/sim.9525 PMC9489667

[35] Cai W van der Laan MJ . One-step targeted maximum likelihood estimation for time-to-event outcomes. Biometrics 2020; 76(3): 722–733. 31729005 10.1111/biom.13172

[36] Mccoy DB Hubbard AE Schuler A , et al. Cross-validated decision trees with targeted maximum likelihood estimation for nonparametric causal mixtures analysis, 2023. https://arxiv.org/abs/2302.07976.

[37] van der Laan M Wang Z van der Laan L . Higher order targeted maximum likelihood estimation, 2021. https://arxiv.org/abs/2101.06290.

[38] Glynn AN Quinn KM . An introduction to the augmented inverse propensity weighted estimator. Cambridge, MA; Cambridge University Press, 2009.

[39] Zhong Y Kennedy EH Bodnar LM , et al. AIPW: an R package for augmented inverse probability–weighted estimation of average causal effects. Am J Epidemiol 2021; 190(12): 2690–2699. 34268567 10.1093/aje/kwab207 PMC8796813

[40] Schnitzer ME Cefalu M . Collaborative targeted learning using regression shrinkage. Stat Med 2018; 37: 530–543. 29094375 10.1002/sim.7527

[41] Zivich PN Breskin A . Machine learning for causal inference: on the use of cross-fit estimators. Epidemiology 2021; 32(3): 393–401. 33591058 10.1097/EDE.0000000000001332 PMC8012235

[42] van der Laan MJ Gruber S . Collaborative double robust targeted maximum likelihood estimation. Int J Biostat 2010; 6(1): 17. 10.2202/1557-4679.1181 PMC2898626 20628637

